# Histone Deacetylases Play a Major Role in the Transcriptional Regulation of the *Plasmodium falciparum* Life Cycle

**DOI:** 10.1371/journal.ppat.1000737

**Published:** 2010-01-22

**Authors:** Balbir K. Chaal, Archna P. Gupta, Brigitta D. Wastuwidyaningtyas, Yen-Hoon Luah, Zbynek Bozdech

**Affiliations:** School of Biological Sciences, Nanyang Technological University, Singapore; Albert Einstein College of Medicine, United States of America

## Abstract

The apparent paucity of molecular factors of transcriptional control in the genomes of *Plasmodium* parasites raises many questions about the mechanisms of life cycle regulation in these malaria parasites. Epigenetic regulation has been suggested to play a major role in the stage specific gene expression during the *Plasmodium* life cycle. To address some of these questions, we analyzed global transcriptional responses of *Plasmodium falciparum* to a potent inhibitor of histone deacetylase activities (HDAC). The inhibitor apicidin induced profound transcriptional changes in multiple stages of the *P. falciparum* intraerythrocytic developmental cycle (IDC) that were characterized by rapid activation and repression of a large percentage of the genome. A major component of this response was induction of genes that are otherwise suppressed during that particular stage of the IDC or specific for the exo-erythrocytic stages. In the schizont stage, apicidin induced hyperacetylation of histone lysine residues H3K9, H4K8 and the tetra-acetyl H4 (H4Ac4) and demethylation of H3K4me3. Interestingly, we observed overlapping patterns of chromosomal distributions between H4K8Ac and H3K4me3 and between H3K9Ac and H4Ac4. There was a significant but partial association between the apicidin-induced gene expression and histone modifications, which included a number of stage specific transcription factors. Taken together, inhibition of HDAC activities leads to dramatic de-regulation of the IDC transcriptional cascade, which is a result of both disruption of histone modifications and up-regulation of stage specific transcription factors. These findings suggest an important role of histone modification and chromatin remodeling in transcriptional regulation of the *Plasmodium* life cycle. This also emphasizes the potential of *P. falciparum* HDACs as drug targets for malaria chemotherapy.

## Author Summary

*Plasmodium falciparum*, a parasitic protozoan, causes the most lethal form of human malaria, killing more than 2 million people per year. It has a complex life cycle that involves distinct morphological stages accompanied by stage specific gene expression in both the mosquito and human hosts. The lack of a vaccine for malaria and widespread resistance highlights the urgency for new anti-malarial drugs that act on different parasite targets. We show that inhibition of histone deacetylase activities results in activation and repression of transcriptionally regulated genes in multiple stages of the *P. falciparum* asexual life cycle. We also show that inhibition disrupts the steady-state level of histone acetylation and methylation across the *P. falciparum* genome. Our data strongly implies that in *P. falciparum*, inhibition of histone deacetylase activity leads to a dramatic increase in global acetylation of histones and subsequently disruption of stage specific gene expression. This process then leads to a collapse of the transcriptional cascade of *P. falciparum*. Therefore, the essential role of histone deacetylases in *Plasmodium* parasites suggests their high potential as molecular targets for malaria intervention strategies.

## Introduction

Gene expression in the asexual intraerythrocytic developmental cycle (IDC) of *Plasmodium falciparum* and *vivax* occurs in a continuous cascade with the induction of most genes occurring just once in the cycle, presumably at the time when their products are required [1],[2],[3]. The next obvious and intriguing step is to understand how this highly specialized mode of transcriptional regulation is controlled. Emerging evidence from other eukaryotes indicates that chromatin structure regulates gene expression through histone modifications such as acetylation, deacetylation and methylation [4].

Histone acetyltransferases (HATs) catalyze acetylation of lysine residues located within histones, thereby reducing chromatin compaction and making the DNA more accessible to regulatory proteins resulting in transcriptional activation. The removal of acetyl groups from the lysine residues is catalyzed by histone deacetylases (HDACs) resulting in chromatin condensation and transcriptional repression. Specific recruitment of HAT and HDAC containing complexes to selected promoter elements generate localized domains of modified histones that influence transcriptional activity [5],[6]. HATs and HDACs also function globally throughout the genome resulting in a highly dynamic equilibrium of histone acetylation and deacetylation reactions, which maintains a steady-state level of histone acetylation across the entire genome [7],[8].

The HDAC super-family is grouped into different classes according to sequence similarity to yeast prototypes. Classes I, II and IV are related to the zinc-dependent yeast Rpd3 or Hda1 deacetylases [9]. Class III HDACs, are a family of NAD-dependent sirtuins related to the yeast silencing information regulator 2 (Sir2) which mediates gene silencing at telomeres, mating-type loci and ribosomal DNA [10]. Homologues of a Class I and Class III HDAC, referred to as PfHDAC1 (PFI1260c) and PfSir2 (PF13_0152) respectively, have been characterized in *P. falciparum* [11],[12]. Transcripts of the nuclear localized PfHDAC1 [11] were detected throughout the asexual IDC and in the exo-erythrocytic stages [2]. The PfSir2 co-localizes with telomeric clusters generating heterochromatin at the chromosome ends. In addition, PfSir2 binding and deacetylation controls the mutual exclusive expression of the surface antigen family encoded by the telomeric *var* genes [12]. The genome sequence of *P. falciparum* has revealed three additional HDAC homologues (PF14_0489, PF14_0690 and PF10_0078) all of which have yet to be characterized [13].

To understand the regulation of gene expression in *P. falciparum* we took the advantage of HDAC inhibitors altering gene transcription [14]. Recently, FR235222, a cyclic tetra-peptide was shown to inhibit HDAC3 activity in *Toxoplasma gondii*, resulting in nucleosomal hyperacetylation of histone 4 [15]. The Class I and II HDAC inhibitor apicidin, also a cyclic tetra-peptide, exhibits anti-proliferative activity against several Apicomplexan parasites including *P. falciparum* [16] and inhibits the enzymatic activity of PfHDAC1 [17]. We showed that apicidin caused profound transcriptional changes in multiple stages of the *P. falciparum* IDC that were characterized by specific induction of genes that are otherwise suppressed during that particular stage. We also showed that apicidin induced rapid hyperacetylation of histone lysine residues associated with promoters and coding regions of a large number of genes indicating the disruption of both targeted and non-targeted HDAC activities. Interestingly, only a partial overlap between the genetic loci with altered histone modifications and genes induced/repressed by apicidin was found. Intriguingly, induction of stage specific transcription factors and other transcription associated or chromatin binding proteins was identified. The significant enrichment of the apicidin induced histone modifications in these gene classes suggests their role in the transcriptional de-regulation in the apicidin treated cell. These findings highlight the important role of histone deacetylases in the transcriptional regulation of the *Plasmodium* life cycle.

## Results

### Activation and repression of transcriptional regulated genes by a HDAC inhibitor

A Class I and II HDAC inhibitor, apicidin, was used to analyze the global transcriptional response of *P. falciparum* to inhibition of its histone deacetylase activities. For this purpose, highly synchronized *P. falciparum* cells were treated with 70nM apicidin at the three asexual developmental stages: ring (6–14 hours post invasion (hpi)), trophozoite (20–28 hpi) and schizont (34–42 hpi). Treatment of *P. falciparum* cells with 70nM of apicidin at these three developmental stages resulted in ∼90% reduction of growth (IC90) that was monitored by the number of newly formed rings after the completion of the IDC (data not shown). To capture the dynamics of the transcriptional response, RNA samples were collected at 0.5, 1, 2, 4 and 6 hours post treatment and analyzed by the *P. falciparum* DNA microarray that represents 5363 coding sequences [18] (Dataset S1).

**Figure 1:**
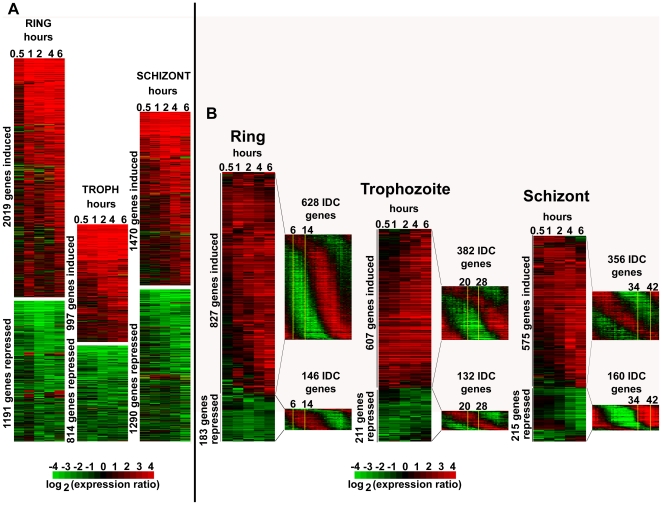
Global transcriptional response of *P. falciparum* to apicidin treatment. Highly synchronized *P. falciparum* cells: rings (6–14 hpi), trophozoites (20–28 hpi) and schizonts (34–42 hpi) were treated with DMSO (0.005%) and apicidin (IC90). RNA samples were collected at 0.5, 1, 2, 4 and 6 hours post treatment. cDNA, synthesized from the RNA samples, was labeled with Cy5 and hybridized against the Cy3 labeled 3D7 reference pool. The microarray data, which included mRNA abundance ratios between each time point sample and the 3D7 reference pool, was filtered as described in material and methods. A. For all stages, genes with a 2 or greater fold difference in expression induced by apicidin treatment in at least one time point are shown. B. For rings and trophozoites, genes with a 3 or greater fold difference in expression in at least two or one time points respectively are shown. For schizonts, genes with a 4 or greater fold difference in expression in at least one time point are shown. The transcription profile of the genes under normal developmental conditions is shown in the *P. falciparum* IDC transcriptome. The stage of the parasites used in the time course experiment is shown boxed in yellow.

Overall, we observed highly dynamic transcriptional changes induced by apicidin in all the three developmental stages (Figure 1A). In particular, 6 hours of apicidin treatment altered the expression of 3210 (59.8% of the genome), 1811 (33.8%) and 2760 (51.5%) genes by at least 2-fold in the ring, trophozoite and schizont stages, respectively. Intriguingly, approximately half of these genes were induced or repressed as early as 1-hour post treatment. To our knowledge, this is one of the most dramatic perturbations of the *P. falciparum* IDC transcriptome reported so far. This is in sharp contrast with previous studies showing non-specific and low amplitude changes in mRNA abundance induced by the anti-malarial drug chloroquine [19] and the antifolate WR99210 [20]. Interestingly, the rapid and large changes in gene expression observed by inhibition of HDAC activities also contradict that of inhibition of *P. falciparum* HAT activities [21].

To understand the physiological relevance of the HDAC-dependent transcriptional response we carried out functional enrichment analyses of the genes induced/repressed by apicidin in all the three developmental stages. Using three types of functional terms Gene Onthology (GO), Kyoto Encyclopedia of Genes and Genomes (KEGG) and Malaria Parasite Metabolic Pathways (MPMP), we identified a large number of functional groups that are significantly enriched in the apicidin altered gene expression (Figure S1). The vast majority of the identified gene groups represented basic metabolic and cellular functions expressed during the *P. falciparum* IDC. In the case of apicidin induced gene expression, a considerate overlap was found among the gene groups associated with the three different stages of the IDC. These included “Fatty acid synthesis in the apicoplast” (P value <0.008) and “Molecular motor prototypes” (P value <0.004). In contrast, with apicidin induced gene repression distinct functional groups were statistically overrepresented in each stage. This can be explained by the fact that any gene repression should be observed in genes that are under normal growth conditions expressed during that particular stage of apicidin treatment. For example, gene groups associated with transcription and translation that are normally induced during the ring stage were significantly repressed (P value <0.01) during this stage [1]. Similarly, gene groups associated with invasion that are normally induced during the schizont stage were significantly repressed (P value <10^−9^) during this stage.

To investigate whether apicidin induced a specific de-regulation of the *P. falciparum* IDC transcriptional cascade [1], we focused on genes with the most pronounced changes (>3–4 fold) in their transcript levels (Figure 1B). In all three stages, this analysis revealed that essentially most of the genes induced by apicidin are under normal growth conditions suppressed during that particular stage of treatment (Figure 1B). For example, genes associated with the TCA cycle that are normally expressed during the trophozoite and early schizont stage were significantly induced (P value = 3.25×10^−3^) in the ring stage. Similarly, genes belonging to the functional group “Subcellular localization of proteins involved in invasion” that are normally expressed in schizonts, were strongly induced (P value = 1.04×10^−3^) in the trophozoite stage by apicidin.

Using the publicly available *P. falciparum* exo-erythrocytic transcriptome data [2], we also examined the top 500 genes with the highest mRNA abundance in the sporozoite and gametocyte stages. A significant number of genes that are considered specific for theses stages and are normally suppressed during the IDC were induced by apicidin (Figure S2). These included genes encoding the early gametocyte markers, Pfg27, Pfs16, Pfpeg-3 and Pfpeg-4 [22], Pf47: a member of the Pfs48/45 gene family [23] and Pfg377: localized in the osmiophilic bodies of gametocytes [24]. Similarly genes associated with sporozoite invasion such as circumsporozoite protein, sporozoite surface protein 2, SPATR and Pf52 [25] were significantly induced (>2-fold) by apicidin.

Taken together, apicidin, a specific inhibitor of HDAC activities affected expression of approximately half of the genome during the *P. falciparum* IDC. Therefore, as a result, a large number of functional groups were significantly affected. The induced expression of stage specific genes indicates that the changes in mRNA abundance are not compatible with arrest of the IDC, but rather a generic de-regulation of the transcriptional cascade of the *P. falciparum* life cycle. Intriguingly, with 70nM of the HDAC inhibitor, this de-regulation is highly dynamic affecting a large number of genes as early as 1-hour post treatment.

### Global levels of histone 3 and 4 acetylation altered by apicidin

**Figure 2:**
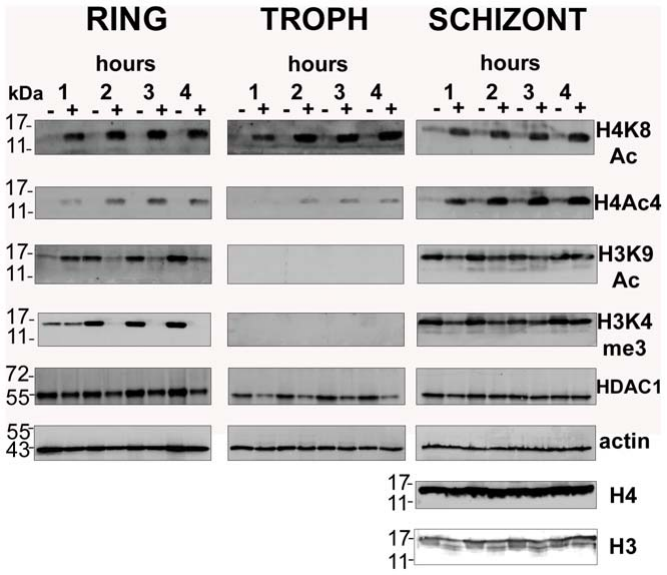
Global histone hyperacetylation induced by apicidin. *P. falciparum* cells were treated with DMSO (0.005%) and apicidin (IC90). Protein samples collected at 1, 2, 3 and 4 hours post treatment were analyzed by SDS-PAGE. PfHDAC1, Pfactin 1, core histone 3 (H3), core histone 4 (H4) and the H4K8Ac, H4Ac4, H3K9Ac and H3K4me3 sites were detected by immunodetection. Molecular weights are shown in kDa. − and + refers to DMSO and apicidin treated cells respectively.

To investigate the mechanisms by which apicidin deregulates the *P. falciparum* transcriptional cascade we examined the effect of this HDAC inhibitor on histone modifications. Previous studies have showed that the HDAC inhibitors Trichostatin A (TSA) and the two derivatives of 2-aminosuberic acid induce overall acetylation levels of *P. falciparum* histone 4 [26]. In *P. falciparum*, previous studies have found stage-specific enrichment of acetylation at histone 3 lysine 9 at putative transcriptional initiation sites, corresponding to stage-specific expression of genes [27]. Therefore, we tested the effect of apicidin on the overall levels of three distinct acetylations: histone 3 lysine 9 (H3K9Ac), H4K8Ac and histone 4 acetylated on lysine residues 5, 8, 12 and 16 (H4Ac4) (Figure 2). In addition, we also tested for tri-methylation of histone 3 lysine 4 (H3K4me3) which has been linked with gene expression [28].

The most striking observation was the dramatic increase in the levels of H4K8Ac and H4Ac4 in the apicidin treated *P. falciparum* cells in all three stages. This strongly indicates that the histone acetylase and deacetylase activities specific for these histone 4 modifications exist in a highly dynamic equilibrium throughout the entire IDC. The increase in histone 4 acetylation observed as early as 1-hour post treatment coincides well with the rapid transcriptional changes induced by apicidin.

Conversely, apicidin induced a different effect on both H3K9Ac and H3K4me3. In rings, a dramatic decrease in the levels of both histone 3 modifications was observed after 2 hours of apicidin treatment. In the first hour of treatment, an increase or no change in H3K9Ac and H3K4me3 respectively was found. In contrast to rings, no change in the overall levels of both modifications was detected in schizonts. In trophozoites these modifications were undetected (Figure 2). In agreement with a previous study [29], immunodetection of core histones 3 and 4 generated an extremely weak signal in both rings and trophozoites (data not shown). Therefore, Pfactin 1 (PFL2215w) was used as a loading control for these two stages (Figure 2). In schizonts, core histones 3 and 4 as well as actin served as suitable loading controls. Interestingly protein levels of PfHDAC1, a target of the HDAC inhibitor, were not altered by the apicidin treatment.

Taken together, we observed different types of histone modifications induced by the HDAC inhibitor apicidin. While an increase in H4K8Ac and H4Ac4 was observed in all stages, the two histone 3 modifications exhibited a more complex response to HDAC inhibition. These results indicate the existence of distinct mechanisms that control the global levels of histone modifications and are directly or indirectly associated with HDAC activities.

### Inhibition of HDACs disrupts the steady-state level of histone acetylation and methylation across the *P. falciparum* genome

Although the western blot analyzes indicated the effect of apicidin on nucleosomal histone modifications, in our next step we wished to investigate the distribution of these induced modifications along the *P. falciparum* genome. The main goal was to identify chromosomal regions that are under the regulatory control of Class I and II HDACs.

**Figure 3:**
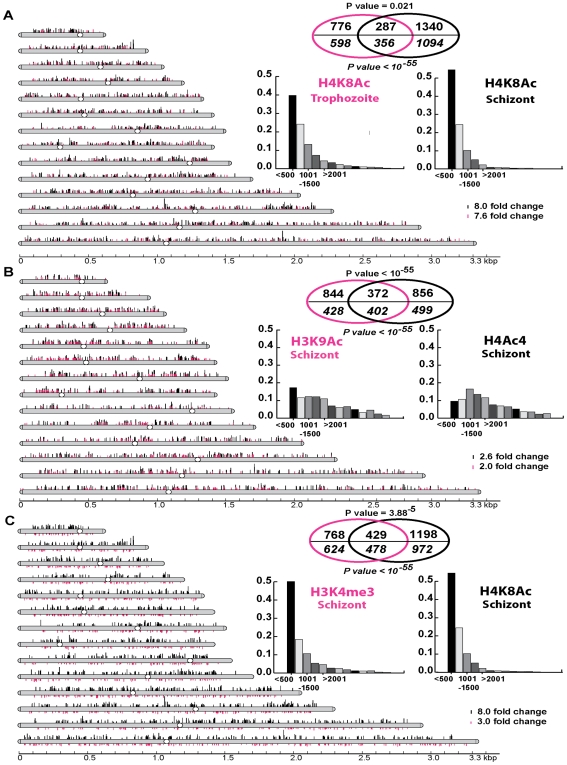
Genome-wide histone hyperacetylation induced by apicidin. A. Chromosomal display of genetic loci, represented by MOEs, that showed a significant increase in H4K8Ac in apicidin treated trophozoites (Δ = 1.7) and schizonts (Δ = 5). B. Chromosomal display of genetic loci that showed a significant increase in H3K9Ac (Δ = 2) and H4Ac4 (Δ = 3.5) in apicidin treated schizonts. C. Chromosomal display of genetic loci that showed a significant *decrease* in H3K4me3 (Δ = 4) and increase in H4K8Ac (Δ = 5) in apicidin treated schizonts. The position of each line reflects the location of each genetic loci within the *P. falciparum* 14 chromosomes. Venn diagrams show significant overlaps between different groups of genetic loci (shown above the line) and genes (shown in *italics* below the line) associated with apicidin altered histone modifications. P values for genetic loci and genes are shown above and below the Venn diagrams respectively. The insert graphs show the distribution of the enriched histone modifications within individual genes (bins of 500 base pairs). MOEs were divided into groups according to their distance from the putative ATG start codon.

Thus, we carried out chromatin immunoprecipitations in conjunction with microarray analysis (ChIP-chip) using antibodies against the four histone modifications H4K8Ac, H4Ac4, H3K9Ac and H3K4me3. Using this approach, we compared changes in the chromosomal distribution of these modifications in *P. falciparum* cells after 1 hour of apicidin treatment (Figure 3). For the ChIP-chip analysis, we used the *P. falciparum* long oligonucleotide DNA microarray, utilized for the transcriptional analyzes, which represents 5363 coding sequences and features one microarray oligonucleotide element (MOE) per 1198 base pairs on average for all the *P. falciparum* coding sequences [18]. To ensure the statistical significance of the obtained data, each ChIP-chip assay was conducted in triplicate and the chromosomal regions with apicidin induced modifications were identified using the method Significance Analysis of Microarrays (SAM) [30] (see materials and methods). To evaluate significant overlaps between the different histone modifications we utilized several different SAM threshold cutoffs (Δ) non of which, however, yielded data with a false discovery rate (FDR) greater than 0.11% (supporting information).

First, we identified 1063 and 1627 genetic loci (represented by the MOEs) that showed a significant increase in H4K8Ac in apicidin treated trophozoites and schizonts respectively (Figure 3A). With an overlap of 287 loci, a negative correlation (P value = 0.021) between these two groups was found. However, when overlapping genes instead of genetic loci, a most striking positive correlation (P value <10^−55^) with an overlap of 356 genes was found. This indicates that, although, increased H4K8Ac in apicidin treated trophozoites and schizonts occurred in similar genes, the increase occurred at different loci within the same gene in the two stages. This is consistent with the model that each developmental stage is characterized by its distinct pattern of histone modifications that undergo progressive changes along the IDC [29],[31].

In addition, in the schizont stage we identified 1216 and 1228 genetic loci that showed a significant increase in H3K9Ac and H4Ac4, after 1 hour of apicidin treatment, respectively (Figure 3B). Overlapping these two groups of genetic loci (372 MOEs) showed a positive correlation (P value <10^−55^) between H3K9Ac and H4Ac4. Moreover, we identified 1197 genetic loci that showed a significant *decrease* in H3K4me3 in apicidin treated schizonts (Figure 3C). The overlap of 429 MOEs between the two groups of genetic loci associated with decrease of H3K4me3 and increase of H4K8Ac (schizonts) showed a positive correlation (P value = 3.88×10^−5^) between these two modifications. Similarly, when overlapping genes instead of genetic loci, positive correlations (P value <10^−55^) between H3K9Ac and H4Ac4 (Figure 3B) as well as H4K8Ac and H3K4me3 (Figure 3C) were observed.

However, these results are not reflected in the western blot analyzes which showed dramatic changes in the overall levels of H4Ac4/H4K8Ac but no change in H3K9Ac/H3K4me3 in apicidin treated schizonts. This apparent discrepancy can be explained by higher amplitudes of apicidin induced H4Ac4/H4K8Ac compared to H3K9Ac/H3K4me3. Hence, these data suggests that despite the differences in their overall response to apicidin treatment across the *P. falciparum* IDC there is some level of association between H4Ac4 and H3K9Ac aswell as H4K8Ac and H3K4me3 in the schizont stage.

Beside these two positive correlations, the majority of genetic loci associated with increased H4K8Ac (both in trophozoites and schizonts) and decreased H3K4me3 originate from the first 500 base pairs of the gene coding sequences. In contrast, the genomic regions associated with H3K9Ac and H4Ac4 exhibit essentially no positional preference within the individual genes (Figure 3, insert graphs). Interestingly, genetic loci associated with H4K8Ac and/or H3K4me3 showed a tendency to be mutually exclusive to loci associated with H4Ac4 and/or H3K9Ac (supporting information).

In summary, these results suggest that HDAC activities in *P. falciparum* counteract at least two chromatin-remodeling pathways. One of these is acetylation at H4K8 and de-methylation of H3K4me3 mainly in the 5′ (possibly promoter) regions of genes. The other pathway is acetylation at H3K9 and tetra-acetylation of histone 4 (at lysine residues 5, 8 12 and 16) which are distributed evenly throughout the coding sequences of *P. falciparum* genes. These two pathways appear to target two largely non-overlapping set of *P. falciparum* genes. It is also important to note that all four of the apicidin induced histone modifications occurred almost exclusively in the intrachromosomal regions of the *P. falciparum* genome. This suggests that both Class I and II HDACs act mainly in the intrachromosomal regions and do not overlap with the activity of PfSir2, the *P. falciparum* Class III HDAC, which generates heterochromatin at the chromosome ends [12].

### Apicidin induces histone hyperacetylation and demethylation along promoter regions of *P. falciparum* genes

**Figure 4:**
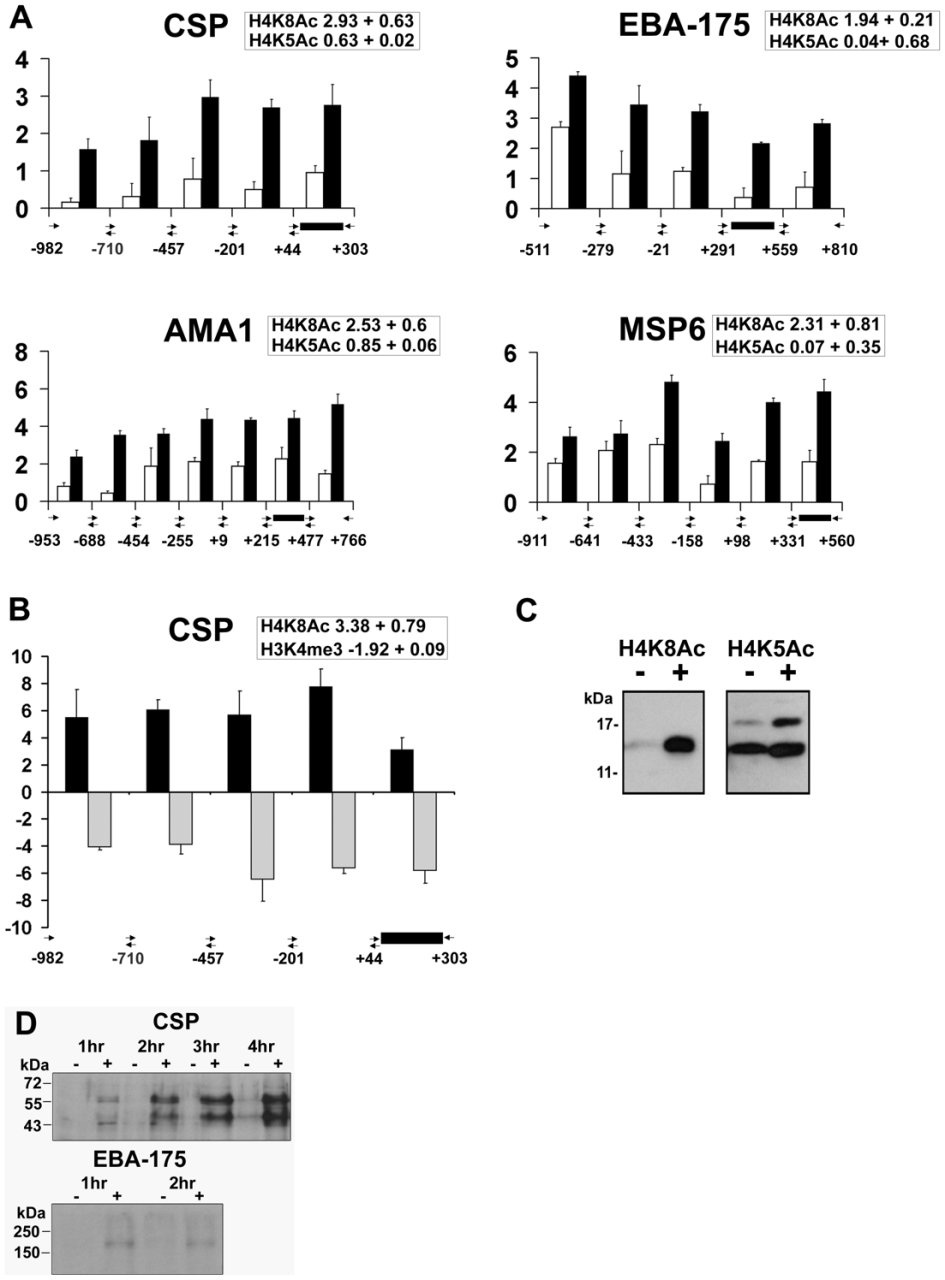
Apicidin induces histone hyperacetylation along promoter regions and protein expression of *P. falciparum* genes. A. Distribution of acetylated H4K8 and H4K5 along the 5′ flanking upstream and coding regions. CHIPs were carried out with trophozoites treated with DMSO (0.005%) and apicidin (IC90) for 1 hour and with antibodies directed against either H4K8Ac or H4K5Ac. B. Distribution of H4K8Ac and H3K4me3 along the 5′ flanking upstream and coding regions of CSP. CHIPs were carried out with schizonts treated with DMSO (0.005%) and apicidin (IC90) for 1 hour and with antibodies directed against either H4K8Ac or H3K4me3. RTQ-PCR was carried out on immunoprecipated DNA and input genomic DNA obtained from DMSO and apicidin treated cells. The log_2_ ratios of H4K8Ac (black bars) and H4K5Ac/H3K4me3 (white/gray bars) were calculated by using the ΔΔCt method (Ct of apicidin-treated immunoprecipitated target gene - Ct of apicidin-treated input target gene) minus (Ct of DMSO treated immunoprecipitated target gene-Ct of DMSO treated input target gene). Black box represents PCR fragment encoding the MOE. The insert boxes show the fold increase in H4K8Ac/H4K5Ac/H3K4me3 in apicidin treated samples, obtained from ChIP-chip data analysis. C and D. Trophozoites were treated with apicidin (IC90) for 1 hour. Protein samples were analyzed by SDS-PAGE followed by immunodetection using an antibody directed against H4K8Ac and H4K5Ac (C) or CSP and EBA-175 (D). Molecular weights are shown in kDa. − and + refers to DMSO and apicidin treated cells respectively.

To further explore the distribution of the apicidin induced histone modifications along the *P. falciparum* genes, we carried out RTQ-PCR measurements with the ChIP immunoprecipitated DNA. Here we focused on four genes that were associated with increased H4K8Ac, in trophozoites treated with apicidin (IC90) for 1 hour. These included circumsporozoite protein (*csp*: PFC0210c), erythrocyte binding antigen 175 (*eba175*: MAL7P1.176), apical membrane antigen 1 (*ama1*: PF11_0344) and merozoite surface protein 6 (*msp6*: PF10_0346) (Figure 4A). In agreement with our ChIP-chip data (Figure 4A insert boxes), significant changes in H4K8Ac were detected within the 5′ extremes of the coding regions of all four genes. In all of the genes, we also detected significant increase in H4K8Ac in the regions upstream to the coding sequence. Overall, we do not detect any consistent bias towards the promoter or the 5′ regions of the coding sequences, which suggests that apicidin induced H4K8Ac spans both of these gene sections evenly. In this study, we compared the apicidin induced H4K8Ac with H4K5Ac whose levels in *P. falciparum* are not significantly affected by this inhibitor (Figure 4C and data not shown).

In addition, we investigated the inverse relationship between H4K8Ac and H3K4me3 that was observed previously in the schizont stage (Figure 3C). In agreement with the ChIP-chip data (insert box), the inverse relationship between these two modifications is preserved in both the 5′ coding regions as well as the upstream non-coding regions of CSP (Figure 4B). Overall, the RTQ-PCR results correlated well with the ChIP-chip data indicating that the observed apicidin induced H4K8 hyperacetylation and H3K4 demethylation occurred in the promoter regions as well as the extreme 5′ regions of *P. falciparum* genes.

In addition to their transcriptional up-regulation, both CSP and EBA-175 proteins were detected in apicidin treated trophozoites (Figure 4D). CSP is N-terminally processed during sporozoite invasion of hepatocytes, resulting in a processed product that is 8–10 kD smaller [32]. Interestingly, we detected two molecular weight forms of CSP: the lower band migrates at the expected molecular weight of the CSP processed product, which suggests the presence of the putative protease in these cells. Increased protein levels of CSP, both processed and unprocessed, were also observed in apicidin treated rings and schizonts (data not shown); this was consistent with the drug induced gene expression of CSP found in all three stages of the IDC. These data indicate the likelihood that transcripts induced by apicidin are also translated. This suggests a lack of translational “checkpoints” in stage specific protein expression and re-emphasizes the importance of transcriptional control for this process.

### Inhibition of HDACs induces expression of transcription associated proteins

To investigate the association between genetic loci with altered histone modifications and genes induced/repressed by apicidin we determined the correlation between the RNA expression and ChIP-chip data obtained with apicidin treated schizonts. In particular, we analyzed the overlap between gene sets induced/repressed by apicidin, in each individual time point and gene sets with altered histone modifications (Tables 1 and 2). For this analysis, we used identical SAM threshold cutoffs as described in Figure 3.

**Table 1:** Association between the genetic loci with altered histone modifications and genes induced by apicidin.

**Hours**	**0.5**		**1**		**2**		**4**		**6**	
**Genes induced**	422	P value	595	P value	773	P value	970	P value	1124	P value
**H4Ac4 (901)**	74		100		124		153		176	
**H4K8Ac (1450)**	95	*0.0424*	135	*0.0184*	189		253		277	
**H3K9Ac (830)**	52		69	*0.0107*	94	*0.0151*	130		163	
**H3K4me3 (1102)**	76		112		144		194		215	

Genes induced by 2-fold or greater, in each individual time point in apicidin treated schizonts was determined. In brackets the number of genes associated with an apicidin induced histone modification in at least one locus corresponding to an MOE are shown. For each individual time point, the number of genes with both a change in RNA expression (>2-fold) and associated with an apicidin induced histone modification are shown. Blank boxes refer to P values >0.05. P values represented as *italics* correspond to negative correlation.

**Table 2:** Association between the genetic loci with altered histone modifications and genes repressed by apicidin.

**Hours**	**0.5**		**1**		**2**		**4**		**6**	
**Genes repressed**	319	P value	466	P value	514	P value	593	P value	737	P value
**H4Ac4 (901)**	50		85		86		103		127	
**H4K8Ac (1450)**	68	*0.0273*	140		161	**0.0326**	210	**5.6E-06**	259	**9.8E-07**
**H3K9Ac (830)**	64	**0.025**	106	**2.5E-05**	91		95		102	
**H3K4me3 (1102)**	73		119	**0.0074**	128	**0.0143**	150	**0.0051**	168	

Genes repressed by 2-fold or greater, in each individual time point in apicidin treated schizonts was determined. In brackets the number of genes associated with an apicidin induced histone modification in at least one locus corresponding to an MOE are shown. For each individual time point, the number of genes with both a change in RNA expression (>2-fold) and associated with an apicidin induced histone modification are shown. Blank boxes refer to P values >0.05. P values represented as **bold** or in *italics* correspond to positive and negative correlation respectively.

To our surprise, no positive correlation between the studied histone acetylations and apicidin induced gene expression was found. Moreover, in the earlier time-points, both H4K8Ac and H3K9Ac were negatively correlated (P value <0.0424) with increased transcription (Table 1). Conversely, a positive correlation (P value <0.0326) between these two histone modifications and gene repression was found (Table 2). However, these findings are consistent with previous studies that show H4K8Ac, H4K12Ac and H4K16Ac are negatively correlated with increased gene expression in yeast [33]. Since H3K4me3 has been linked with gene expression [28] it was not, surprising that demethylation of H3K4me3 was found to be positively correlated (P value <0.0143) with gene repression (Table 2).

However, despite this partial correlation, the majority of the apicidin induced/suppressed genes did not associate with the four studied histone modifications. This discrepancy can be explained by two working hypotheses that are not mutually exclusive. First, additional histone modifications that act independently or in the context of each other (e.g. histone code) mediate the apicidin induced transcriptional changes. Given that post-translational modifications on over 60 different histone residues have been detected in eukaryotic systems [4], it will be intriguing to pursue further studies that analyze their role in regulation of the *P. falciparum* life cycle. Second, some of the observed transcriptional changes could result from secondary effects where apicidin alters the expression of stage specific transcription factors and other transcription associated or chromatin binding proteins which in turn contributes to the de-regulation of the transcriptional cascade of *P. falciparum*.

These secondary effects can be especially responsible for the transcriptional changes in the later experimental time-points of the 6-hour apicidin time course treatment during which the number of induced/repressed genes increases progressively (Tables 1 and 2).

**Figure 5:**
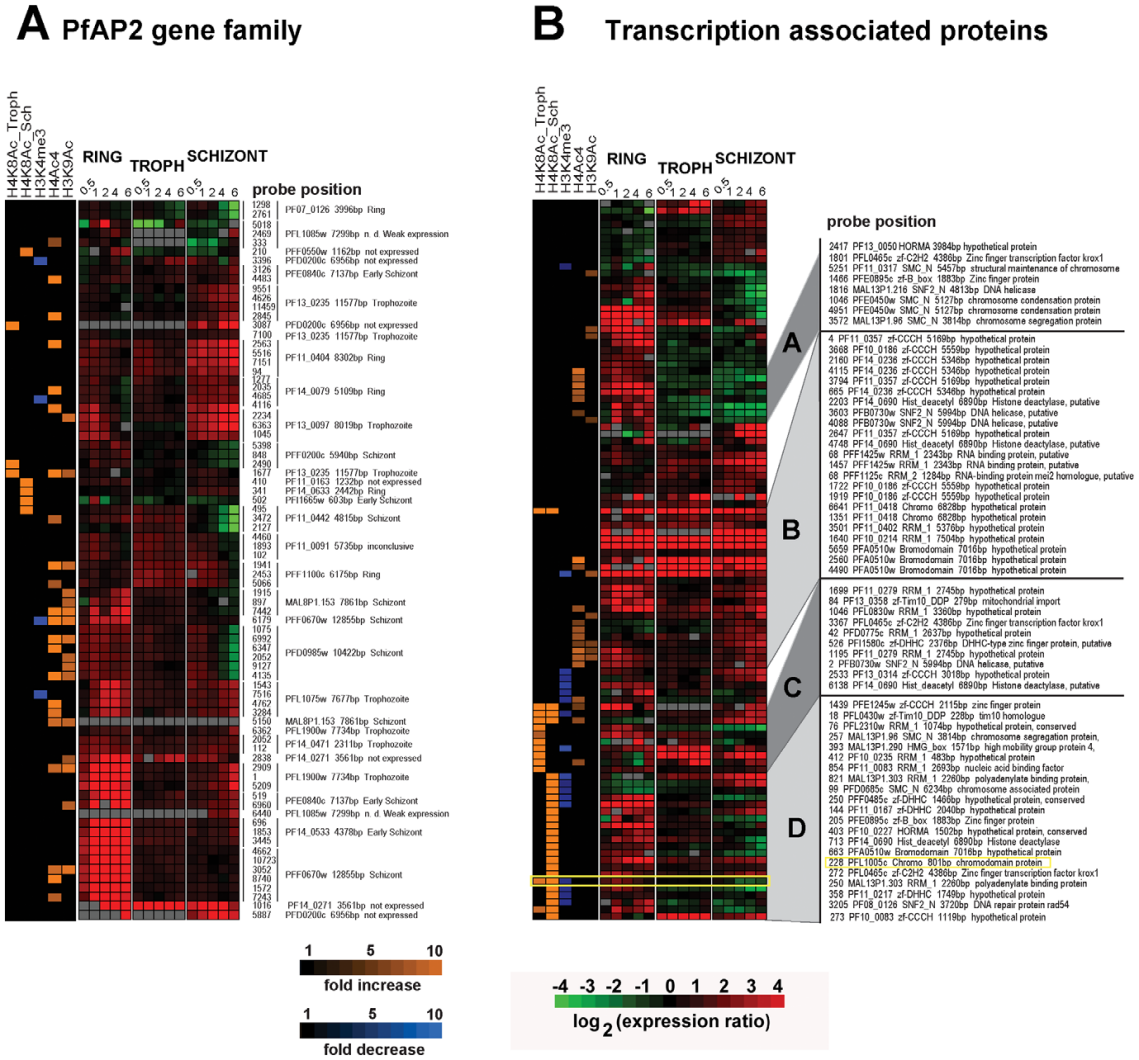
Inhibition of HDACs induces expression of transcription associated proteins. A. Apicidin disrupts stage specific expression of the ApiAP2 proteins. B. Apicidin induces expression of transcription associated proteins. MOEs corresponding to individual genes and showing changes in gene expression and histone modifications induced by apicidin treatment are shown. The specific stage of expression, under normal developmental conditions, for each ApiAP2 protein is indicated on the right hand side. PfHDAC1 (PFI1260c), PfSir2 (PF13_0152), HDAC homologues (PF14_0489, PF14_0690 and PF10_0078), circumsporozoite protein (PFC0210c), erythrocyte binding antigen 175 (MAL7P1.176), apical membrane antigen 1 (PF11_0344), merozoite surface protein 6 (PF10_0346), ApiAP2 proteins (PF14_0633, PFF0200c, PF11_0404, PF14_0079, PF13_0097, PFL1075w, PF14 _0271, PFF1100c and PFD0200c), heterochromatin protein 1 (PFL1005c), Pfg27 (PF13_0011), Pfs16 (PFD0310w), Pfpeg-3 (PFL0795c), Pfpeg-4 (PF10_0164), Pf47 (PF13_0248), Pfg377 (PFL2405c), sporozoite surface protein 2 (PF13_0201), SPATR (PFB0570w), Pf52 (PFD0215c), PfGCN5 (PF08_0034), Pfactin 1 (PFL2215w) and PfMYST (PF11_0192).

Recent reports have suggested that the Apicomplexan AP2 (ApiAP2) family of putative transcriptional regulators play a major role in stage specific gene expression during the *Plasmodium* IDC and possibly other developmental stages. In *P. falciparum*, the ApiAP2 gene family consists of 26 members each of which shows stage specific gene expression spanning the IDC [34]. In addition, two of the ApiAP2 members (PF14_0633 and PFF0200c) were each found to bind specific DNA motifs found in the promoter regions of a number of *Plasmodium* genes with highly coherent expression patterns [35]. In our study, apicidin affected transcription of a number of ApiAP2 genes with the majority of these being up-regulated in the developmental stage in which they are normally suppressed (Figure 5A). To determine association between genetic loci with altered histone modifications and ApiAP2 genes induced by apicidin we overlapped data from RNA expression analysis and ChIP-chip carried out with apicidin treated schizonts. Within 30 minutes of treatment, 8 ApiAP2 genes were significantly induced (>2-fold) and positively correlated (P value = 0.0078) with a change in at least one acetylation or methylation in apicidin treated schizonts. Four of these ApiAP2 proteins, PF11_0404, PF14_0079, PF13_0097 and PFL1075w are normally only expressed during the ring or trophozoite stages. In addition, 3/8 of these ApiAP2 proteins, PF14 _0271, PFF1100c and PFD0200c, classified as not expressed during the asexual erythrocytic stage were also up-regulated in other stages (Figure 5A). It is tempting to speculate that these proteins might be responsible for the expression of the exo-erythrocytic genes that were observed in our study (Figure S2).

In addition, we identified 52 genes that carry at least one predicted domain linked with DNA binding and/or transcriptional regulation and whose expression was affected by apicidin in at least one developmental stage (Figure 5B). Interestingly, 45 out of the 52 genes are also associated with at least one of the apicidin induced histone modifications in at least one locus corresponding to an MOE. We were unable to find a statistically significant correlation between their change in RNA expression and association with a specific apicidin induced histone modification. Although, this is likely due to the heterogeneous character of this compiled group of 52 proteins, some of these factors may be involved in the apicidin induced transcriptional changes.

Hierarchical clustering of the composite dataset revealed 21 genes that are linked with H4K8Ac in the schizont stage while their expression is altered in both schizont and ring stages (Figure 5B cluster D). Interestingly, this cluster includes PFL1005c, recently identified as the heterochromatin protein 1 (PfHP1) that binds to subtelomeric chromatin and is linked to mutually exclusive expression of the major virulence *var* gene family [36],[37]. The sensitivity of *pfhp1* gene expression to apicidin and its association with the H8K4Ac and demethylation of H3K4me3 might signal the link between subtelomeric gene silencing and the general epigenetic regulation in *P. falciparum*. In addition, this cluster contains 9 proteins with transcription factor-like DNA binding domains (*zinc-finger (zf)-B-box*, *zf-CCCH*, *zf-DHHC*, *zf-C2H2*, *zf-Tim10_DDP*, *and SNF2*), 4 proteins with chromatin binding domains (High Mobility Group (*HMG*), structural maintenance of chromosomes (*SMC_N*), and Hop1p, Rev7p and MAD2 (*HORMA*)), and 4 proteins with RNA recognition motif (*RRM*) which were previously implicated in RNA stability, splicing as well as posttranscriptional regulation [38].

Similar functional representation was found in the cluster of genes that are associated with H4K8Ac and over-expressed predominantly in the trophozoite stage (Figure 5B cluster C). Interestingly, one of the HDAC homologues (PF14_0690) is associated with increase in H4K8Ac in apicidin treated trophozoites (Figure 5B cluster C) and schizonts (Figure 5B cluster D). This suggests an intriguing possibility that epigenetic regulation of this gene is mediated by the activity of its own protein product.

The third cluster contains 23 MOE representing 11 genes which are up-regulated by apicidin in one or more developmental stage and are predominantly associated with H4Ac4/H3K9Ac (Figure 5B cluster B). Finally, three proteins with transcription factor-like DNA binding domains and three proteins linked with structural maintenance of chromosomes were found to be associated with H4Ac4 or H3K9Ac and down-regulated in schizonts but up-regulated in rings (Figure 5B cluster A).

In conclusion, we found positive correlation between the apicidin induced histone modifications and gene repression but not with transcriptional activation. However, enrichment of these apicidin induced histone modifications in gene classes associated with transcriptional regulation suggests their role in the observed transcriptional response to the HDAC inhibitor apicidin. Thus, these proteins are suitable candidates as transcriptional regulatory factors during the *P. falciparum* life cycle.

## Discussion

The HDAC inhibitor apicidin induced profound transcriptional changes in all the stages of the *P. falciparum* IDC. Apicidin altered the expression of 59.8%, 33.8% and 51.5% of the genome in the ring, trophozoite and schizont stages respectively (Figure 1). This transcriptional response of *P. falciparum* to the HDAC inhibitor differs considerably from other eukaryotic organisms. Treatment of yeast cells with TSA was found to parallel the expression profile of the yeast *RPD3* deletion strain (Class I HDAC prototype) with only a limited number (200–300) of genes exhibiting altered mRNA levels compared to the wild type strain [39]. Similarly, in cancer cell lines, HDAC inhibitors affect only limited groups of genes that typically include no more than 2–17% of the genome [14],[40].

The vast majority of genes regulated by HDAC inhibitors in mammalian or yeast cells are involved in regulatory functions including apoptosis, mitosis and cell cycle regulation [39],[41]. Surprisingly in *P. falciparum*, we found no bias in gene groups affected by apicidin associated with basic metabolic pathways, cellular pathways or biological processes essential for the parasite's development. Another feature of the apicidin-induced transcriptional response is up-regulation of genes that should be under normal growth conditions only expressed in the following or preceding developmental stage or during the exo-erythrocytic stages (sporozoites and gametocytes). These findings conclude that histone deacetylase activities, that are inhibited by apicidin, play an essential role in regulation of stage specific gene expression in *Plasmodium* parasites.

Apicidin also induced rapid histone hyperacetylation in *P. falciparum* parasites. The *P. falciparum* HATs PfGCN5, involved in acetylation of H3K9 and H3K14 [21], and PfMYST, implicated in acetylation of H4K5, K8, K12 and K16 [42] are ideal candidates to consider for the robust histone hyperacetylation observed by the apicidin treatments. The apicidin-induced histone acetylations and transcriptional changes, observed as soon as 1 hour of treatment, shows the highly dynamic role of HDAC and HAT activities in chromatin remodeling and its profound effect on transcriptional regulation.

Work by Vogelauer et al [7] showed that deletion of Class I and II HDACs resulted in HAT induced global histone acetylation, spanning large chromosomal regions containing both intergenic and coding regions. They also showed that, promoter targeted histone modifications occur in a background of global acetylation and deacetylation that exists in a dynamic equilibrium across the genome controlling basal transcription. This was further explored by Katan-Khaykovich and Struhl [8] who showed that reversal of targeted histone deacetylation or acetylation, upon dissociation of a repressor or activator, to the initial state of acetylation was carried out rapidly by the non-targeted global acetylation or deacetylation respectively. Reversal of targeted acetylation, by globally acting HDACs, was found to be 3–5 times more rapid than that of targeted deacetylation. In agreement with this model, our data strongly implies that in *P. falciparum* inhibition of HDAC activity leads to a dramatic increase in global acetylation of histones and subsequently a general induction of basal transcription. This process then leads to a collapse of the transcriptional cascade of the *P. falciparum* IDC.

Interestingly, only a partial overlap between the genetic loci with altered histone modifications and genes induced/repressed by apicidin was observed. Why did some genes respond transcriptionally to HDAC inhibition while others did not? Previous studies have shown that in spite of global hyperacetylation of core nucleosomal histones induced by HDAC inhibitors, this does not result in global changes in gene expression [40]. Specific histone modifications recruit binding of non-histone proteins such as transcription factors [43],[44], chromatin remodeling proteins [36],[45] and complexes [43]. Therefore particular combinations of histone modification patterns can dictate specific biological functions such as gene transcription or silencing [46]. Presumably, treatment with HDAC inhibitors would severely alter these histone modification patterns resulting in changes in the biological readout. This emphasizes the intricate and multifaceted processes that control gene expression.

In agreement with our ChIP-chip analysis, Lopez-Rubio et al [47] have also showed that H3K4me3 has a broad distribution across the *P. falciparum* genome and is mainly absent from the heterochromatin loci found at the chromosome ends. An interesting finding of this perturbation study has been the decrease in levels of nucleosomal H3K4me3 (Figure 3C and 4B). This histone modification, in particular, is intriguing since it recruits both activating *and* repressing effector proteins and complexes such as the yeast SAGA complex, which contains the GCN5 HAT, and the Sin3-HDAC1 deacetylation complex [48]. How the inhibition of HDAC activities results in decreased levels of H3K4me3 is yet unclear.

ChIP-chip analysis of the knockout strain for the PfSir2 showed that H3K9me3 levels were reduced and H3K9Ac levels were increased, compared to the wild type strain, in the 5′UTR regions of a subset of up-regulated *var* and *rifin* genes [47]. This proposes a link between Sir2-mediated deacetylation and tri-methylation of H3K9 [36]. Similarly, in our findings inhibition of either Class I or II HDAC activities results in reduced H3K4me3 levels on nucleosomes associated with putative promoter sites (Figure 3C and 4B). It will be intriguing to identify any cross-talk between HDACs and tri-methylation of histone 3 lysine 4.

Although the mechanisms of the growth inhibition of *Plasmodium* cells by HDAC inhibitors need further studies, our data suggest that the general de-regulation of the global transcriptional regulation might be one of its important (if not the most important) component. The profound effect of the HDAC inhibitor on *P. falciparum* growth suggests a high potential of HDAC enzymes as molecular targets for malaria intervention strategies [17]. Given the importance of transcriptional regulation in other *Plasmodium* developmental processes such as hepatocyte invasion and schizogony [49] and gametocytogenesis [50], HDAC inhibitors might provide good candidates for chemotherapeutic development for these stages.

## Materials and Methods

### Parasite culture and drug treatment

The *P. falciparum* 3D7 clone was cultured and synchronized as described by Bozdech et al [51]. Apicidin *Fusarium* sp (Calbiochem) was prepared as a stock in 100% di-methyl sulfoxide (DMSO). Synchronized *P. falciparum* cells, at 5% parasitemia and 2% hematocrit, were treated with 70nM apicidin. This concentration of 70nM apicidin represents the median IC90 value determined from the individual IC90 values calculated for each stage of the IDC. The IC90 concentration resulted in 90% reduction of growth (IC90), compared to matched DMSO (0.005%) treated controls, that was monitored by the number of newly formed rings after the completion of the IDC (data not shown). Rings, trophozoites and schizonts were grown in the presence of either DMSO or apicidin for 48, 32 and 18 hours respectively and then examined by Giemsa staining, Cells were harvested by centrifugation at 1,500g for 5 min, washed in phosphate buffered saline (PBS) and pelleted at 1,500g for 5 min. Cell pellets were rapidly frozen in liquid nitrogen and stored at −80°C.

### RNA expression analysis by microarray hybridization

Synchronized *P. falciparum* cells: rings (6–14 hpi), trophozoites (20–28 hpi) and schizonts (34–42 hpi) were treated with either DMSO (0.005%) or 70nM apicidin as described above. Cells were harvested at 0.5, 1, 2, 4 and 6 hours post treatment. Total RNA extraction, amino-allyl cDNA synthesis and DNA microarray was carried out as described previously [51]. cDNA, synthesized from each time point sample, was hybridized against a 3D7 reference pool which was assembled from equal mass of RNA from samples representing 8 hour interval stages throughout the IDC. A *P. falciparum* genome-wide microarray containing 10166 MOEs representing 5363 coding sequences was used for this study [18]. The raw microarray data included mRNA abundance ratios between each time point sample and the 3D7 reference pool. These data were subjected to linear normalization and filtered as follows: signal intensity > background intensity + 2 × standard deviation of background intensity, recorded for each MOE individually. MOE ratios were averaged for genes with more than one MOE (Dataset S1). Hierarchical clustering was carried out using log-transformed ratios in Gene Cluster and visualized using Java Treeview [51].

### Immunodetection of altered histone modifications and induced protein expression

Synchronized ring, trophozoite and schizont stage parasites were treated with either DMSO (0.005%) or 70nM apicidin as described above. Cells collected at 1, 2, 3 and 4 hours post treatment were lyzed in saponin (0.15%) and washed three times with PBS. The isolated parasites were resuspended directly in SDS sample buffer, incubated at 100°C for 10 min and centrifuged at 16,000g for 10 min. The supernatant was analyzed by 15% SDS-PAGE and transferred onto nitrocellulose membranes. Antibodies directed against core histone 4, H3K9Ac, H4K8Ac, H4K5Ac, H4Ac4 and H3K4me3 were obtained from Upstate Biotechnology. Antibodies against core histone 3 and Pfactin 1 were obtained from Abcam (1791) and Sigma (A2066) respectively. Antibodies against the *P. falciparum* CSP (MRA-24) and EBA175 (MRA-2) were obtained from the Malaria Research and Reference Reagent Resource Center. The polyclonal PfHDAC1 antiserum was generated by immunizing rabbits with a peptide corresponding to amino acids 421–435, STTHHLRRKNYDDD, of PfHDAC1 (PFI1260c). The peptide had a N-terminal cysteine added for conjugation purposes (i-DNA Biotechnology). Horseradish peroxidase conjugated secondary antibodies and an enhanced chemiluminescence kit were used according to manufacturer's instructions (GE Healthcare).

### Chromatin immunoprecipitation and quantitative real-time PCR

Synchronized trophozoite and schizont stage parasites were treated with either DMSO (0.005%) or 70nM apicidin. After 1 hour of treatment, the cells were lyzed in saponin (0.15%) and washed three times with PBS. Chromatin was crosslinked by incubating the isolated parasites with formaldehyde to a final concentration of 0.5% for 10 min at room temperature. Immediately after, the parasites were treated with glycine (0.125M final concentration) and washed twice with cold PBS.

For nuclei isolation, parasites were resuspended in buffer A (25mM Tris-HCl pH 7.8, 1mM EDTA, 0.25% Nonidet P-40, protease inhibitor cocktail (Roche), 2mM PMSF), incubated on ice for 1 hour and then lyzed by 200 strokes in a pre-chilled homogenizer. The nuclei were pelleted by centrifugation at 2,300g for 5 min. In order to obtain DNA fragments in the range of 200 to 1000 bp, the nuclei was resuspended in 200 µl of sonication buffer (1% SDS, 10mM EDTA, 50mM Tris, pH 8.0), incubated on ice for 15 min and then sonicated 6 times for 10 seconds at 25% amplitude with 1 min intervals between each pulse (Sonics Vibra Cell). After centrifugation at 16,000g for 10 min the supernatant was diluted 10-fold in ChIP Dilution Buffer (Upstate Biotechnology). An aliquot of 100 µl was kept as input DNA and the remainder was subjected to chromatin immunoprecipitation as described by Upstate Biotechnology ChIP Assay Kit (#26225). For each antibody tested a 1∶200 dilution was used.

An equal volume of immunoprecipitated DNA from DMSO and apicidin treated samples was amplified using random primers [52]. From each sample equal concentrations of the amplified DNA was co-hybridized against an input pool DNA. This input pool DNA was assembled from equal mass of input DNA from apicidin and DMSO treated samples. A *P. falciparum* genome-wide microarray representing 5363 coding sequences was used [18]. The raw microarray data included ratios between the immunoprecipitated sample and the input pool DNA. These data were filtered as follows: signal intensity > background intensity + 2 × standard deviation of background intensity, recorded for each MOE individually. To identify MOEs with statistically significant changes in levels of acetylation and methylation the filtered data was analyzed by the method Significance Analysis of Microarrays (SAM) [30]. SAM assigned a score to each MOE on the basis of change in acetylation or methylation level relative to the standard deviation of repeated measurements for that MOE. Various SAM threshold cutoffs (Δ) were used to generate sets of MOEs showing significant changes for each histone modification (supporting information). For each set the percentage of MOEs identified by chance, the false discovery rate (FDR), was determined as 0.11% or less.

RTQ-PCR were carried out, with immunoprecipated and input DNA obtained from DMSO and apicidin treated cells, using the *Power* SYBR Green PCR Master Mix (Applied Biosystems) according to manufacturer's instructions. The log_2_ ratios were calculated by using the ΔΔCt method (Ct of apicidin-treated immunoprecipitated target gene - Ct of apicidin-treated input target gene) minus (Ct of DMSO treated immunoprecipitated target gene-Ct of DMSO treated input target gene), where Ct is the threshold cycle.

## Supporting Information

Dataset S1 (1.19 MB TXT)

Figure S1 Functional classes affected by apicidin treatment. Functional groups significantly associated (P value <0.05) with induced (A) and repressed (B) expression (>2-fold) by apicidin in all three stages of the *P. falciparum* IDC are shown. The functional annotations based on Malaria Parasite Metabolic Pathways (MPMP) are used. The P values are shown as logarithm values with base 10.
(0.39 MB TIF)

Figure S2 Apicidin treatment alters expression of gametocyte (A) and sporozoite (B) genes in asexual blood stages. Highly synchronized *P. falciparum* cells: rings (6–14 hpi), trophozoites (20–28 hpi) and schizonts (34–42 hpi) were treated with 70nM apicidin. RNA samples were collected at 0.5, 1, 2, 4 and 6 hours post treatment. cDNA, synthesized from the RNA samples, was labeled with Cy5 and hybridized against the Cy3 labeled 3D7 reference pool. The data include mRNA abundance ratios between each time point sample and the 3D7 reference pool. The data was filtered as described in material and methods. The first 500 genes showing the highest mRNA abundance in the publicly available *P. falciparum* gametocyte and sporozoite gene expression datasets [2] were analyzed within our apicidin perturbation dataset. Genes with a 2 or greater fold difference in expression, in at least one time point, in apicidin treated rings, trophozoites or schizonts were shown.
(0.62 MB TIF)

Table S1 The effect of apicidin treatment for 1 hour on four histone modifications (H4K8Ac, H3K9Ac, H4Ac4 and H3K4me3) in the schizont stage was studied. In addition, the effect of apicidin treatment for 1 hour on H4K8Ac in the trophozoite stage was also studied. Chromatin immunoprecipitations in conjunction with microarray analysis (ChIP-chip) were carried out.The method Significance Analysis of Microarrays (SAM) was used to statistically discriminate MOEs showing altered histone modifications induced by apicidin treatment. SAM assigned a score to each MOE on the basis of change in acetylation or methylation level relative to the standard deviation of repeated measurements for that MOE. Different SAM threshold cutoffs were used to generate sets of MOEs showing significant changes for each histone modification. For each set the percentage of MOEs identified by chance, the false discovery rate (FDR), was determined. The significance of the overlap between the different sets of MOEs corresponding to each of the histone modifications was also determined. Overlap numbers and P values shown in red correspond to positive correlation.
(0.02 MB XLS)

## References

[1] Bozdech Z, Llinas M, Pulliam BL, Wong ED, Zhu J (2003). The transcriptome of the intraerythrocytic developmental cycle of Plasmodium falciparum.. PLoS Biol.

[2] Le Roch KG, Zhou Y, Blair PL, Grainger M, Moch JK (2003). Discovery of gene function by expression profiling of the malaria parasite life cycle.. Science.

[3] Bozdech Z, Mok S, Hu G, Imwong M, Jaidee A (2008). The transcriptome of Plasmodium vivax reveals divergence and diversity of transcriptional regulation in malaria parasites.. Proc Natl Acad Sci U S A.

[4] Kouzarides T (2007). Chromatin modifications and their function.. Cell.

[5] Li J, Lin Q, Wang W, Wade P, Wong J (2002). Specific targeting and constitutive association of histone deacetylase complexes during transcriptional repression.. Genes Dev.

[6] Bryant GO, Ptashne M (2003). Independent recruitment in vivo by Gal4 of two complexes required for transcription.. Mol Cell.

[7] Vogelauer M, Wu J, Suka N, Grunstein M (2000). Global histone acetylation and deacetylation in yeast.. Nature.

[8] Katan-Khaykovich Y, Struhl K (2002). Dynamics of global histone acetylation and deacetylation in vivo: rapid restoration of normal histone acetylation status upon removal of activators and repressors.. Genes Dev.

[9] Yang XJ, Seto E (2008). The Rpd3/Hda1 family of lysine deacetylases: from bacteria and yeast to mice and men.. Nat Rev Mol Cell Biol.

[10] Gasser SM, Cockell MM (2001). The molecular biology of the SIR proteins.. Gene.

[11] Joshi MB, Lin DT, Chiang PH, Goldman ND, Fujioka H (1999). Molecular cloning and nuclear localization of a histone deacetylase homologue in Plasmodium falciparum.. Mol Biochem Parasitol.

[12] Freitas-Junior LH, Hernandez-Rivas R, Ralph SA, Montiel-Condado D, Ruvalcaba-Salazar OK (2005). Telomeric heterochromatin propagation and histone acetylation control mutually exclusive expression of antigenic variation genes in malaria parasites.. Cell.

[13] Gardner MJ, Hall N, Fung E, White O, Berriman M (2002). Genome sequence of the human malaria parasite Plasmodium falciparum.. Nature.

[14] Mottet D, Castronovo V (2008). Histone deacetylases: target enzymes for cancer therapy.. Clin Exp Metastasis.

[15] Bougdour A, Maubon D, Baldacci P, Ortet P, Bastien O (2009). Drug inhibition of HDAC3 and epigenetic control of differentiation in Apicomplexa parasites.. J Exp Med.

[16] Darkin-Rattray SJ, Gurnett AM, Myers RW, Dulski PM, Crumley TM (1996). Apicidin: a novel antiprotozoal agent that inhibits parasite histone deacetylase.. Proc Natl Acad Sci U S A.

[17] Patel V, Mazitschek R, Coleman B, Nguyen C, Urgaonkar S (2009). Identification and characterization of small molecule inhibitors of a class I histone deacetylase from Plasmodium falciparum.. J Med Chem.

[18] Hu G, Llinas M, Li J, Preiser PR, Bozdech Z (2007). Selection of long oligonucleotides for gene expression microarrays using weighted rank-sum strategy.. BMC Bioinformatics.

[19] Gunasekera AM, Myrick A, Le Roch K, Winzeler E, Wirth DF (2007). Plasmodium falciparum: genome wide perturbations in transcript profiles among mixed stage cultures after chloroquine treatment.. Exp Parasitol.

[20] Ganesan K, Ponmee N, Jiang L, Fowble JW, White J (2008). A genetically hard-wired metabolic transcriptome in Plasmodium falciparum fails to mount protective responses to lethal antifolates.. PLoS Pathog.

[21] Cui L, Miao J, Furuya T, Fan Q, Li X (2008). Histone acetyltransferase inhibitor anacardic acid causes changes in global gene expression during in vitro Plasmodium falciparum development.. Eukaryot Cell.

[22] Silvestrini F, Bozdech Z, Lanfrancotti A, Di Giulio E, Bultrini E (2005). Genome-wide identification of genes upregulated at the onset of gametocytogenesis in Plasmodium falciparum.. Mol Biochem Parasitol.

[23] Templeton TJ, Kaslow DC (1999). Identification of additional members define a Plasmodium falciparum gene superfamily which includes Pfs48/45 and Pfs230.. Mol Biochem Parasitol.

[24] Alano P, Read D, Bruce M, Aikawa M, Kaido T (1995). COS cell expression cloning of Pfg377, a Plasmodium falciparum gametocyte antigen associated with osmiophilic bodies.. Mol Biochem Parasitol.

[25] Kappe SH, Gardner MJ, Brown SM, Ross J, Matuschewski K (2001). Exploring the transcriptome of the malaria sporozoite stage.. Proc Natl Acad Sci U S A.

[26] Andrews KT, Tran TN, Lucke AJ, Kahnberg P, Le GT (2008). Potent antimalarial activity of histone deacetylase inhibitor analogues.. Antimicrob Agents Chemother.

[27] Cui L, Miao J, Furuya T, Li X, Su XZ (2007). PfGCN5-mediated histone H3 acetylation plays a key role in gene expression in Plasmodium falciparum.. Eukaryot Cell.

[28] Barski A, Cuddapah S, Cui K, Roh TY, Schones DE (2007). High-resolution profiling of histone methylations in the human genome.. Cell.

[29] Miao J, Fan Q, Cui L, Li J, Li J (2006). The malaria parasite Plasmodium falciparum histones: organization, expression, and acetylation.. Gene.

[30] Tusher VG, Tibshirani R, Chu G (2001). Significance analysis of microarrays applied to the ionizing radiation response.. Proc Natl Acad Sci U S A.

[31] Salcedo-Amaya AM, van Driel MA, Alako BT, Trelle MB, van den Elzen AM (2009). Dynamic histone H3 epigenome marking during the intraerythrocytic cycle of Plasmodium falciparum.. Proc Natl Acad Sci U S A.

[32] Coppi A, Pinzon-Ortiz C, Hutter C, Sinnis P (2005). The Plasmodium circumsporozoite protein is proteolytically processed during cell invasion.. J Exp Med.

[33] Kurdistani SK, Tavazoie S, Grunstein M (2004). Mapping global histone acetylation patterns to gene expression.. Cell.

[34] Balaji S, Babu MM, Iyer LM, Aravind L (2005). Discovery of the principal specific transcription factors of Apicomplexa and their implication for the evolution of the AP2-integrase DNA binding domains.. Nucleic Acids Res.

[35] De Silva EK, Gehrke AR, Olszewski K, Leon I, Chahal JS (2008). Specific DNA-binding by apicomplexan AP2 transcription factors.. Proc Natl Acad Sci U S A.

[36] Perez-Toledo K, Rojas-Meza AP, Mancio-Silva L, Hernandez-Cuevas NA, Delgadillo DM (2009). Plasmodium falciparum heterochromatin protein 1 binds to tri-methylated histone 3 lysine 9 and is linked to mutually exclusive expression of var genes.. Nucleic Acids Res.

[37] Flueck C, Bartfai R, Volz J, Niederwieser I, Salcedo-Amaya AM (2009). Plasmodium falciparum heterochromatin protein 1 marks genomic loci linked to phenotypic variation of exported virulence factors.. PLoS Pathog.

[38] Coulson RM, Hall N, Ouzounis CA (2004). Comparative genomics of transcriptional control in the human malaria parasite Plasmodium falciparum.. Genome Res.

[39] Bernstein BE, Tong JK, Schreiber SL (2000). Genomewide studies of histone deacetylase function in yeast.. Proc Natl Acad Sci U S A.

[40] Glaser KB, Staver MJ, Waring JF, Stender J, Ulrich RG (2003). Gene expression profiling of multiple histone deacetylase (HDAC) inhibitors: defining a common gene set produced by HDAC inhibition in T24 and MDA carcinoma cell lines.. Mol Cancer Ther.

[41] Xu WS, Parmigiani RB, Marks PA (2007). Histone deacetylase inhibitors: molecular mechanisms of action.. Oncogene.

[42] Smith AT, Tucker-Samaras SD, Fairlamb AH, Sullivan WJ (2005). MYST family histone acetyltransferases in the protozoan parasite Toxoplasma gondii.. Eukaryot Cell.

[43] Agalioti T, Chen G, Thanos D (2002). Deciphering the transcriptional histone acetylation code for a human gene.. Cell.

[44] Lupien M, Eeckhoute J, Meyer CA, Wang Q, Zhang Y (2008). FoxA1 translates epigenetic signatures into enhancer-driven lineage-specific transcription.. Cell.

[45] Wu LP, Wang X, Li L, Zhao Y, Lu S (2008). Histone deacetylase inhibitor depsipeptide activates silenced genes through decreasing both CpG and H3K9 methylation on the promoter.. Mol Cell Biol.

[46] Munshi A, Shafi G, Aliya N, Jyothy A (2009). Histone modifications dictate specific biological readouts.. J Genet Genomics.

[47] Lopez-Rubio JJ, Mancio-Silva L, Scherf A (2009). Genome-wide analysis of heterochromatin associates clonally variant gene regulation with perinuclear repressive centers in malaria parasites.. Cell Host Microbe.

[48] Berger SL (2007). The complex language of chromatin regulation during transcription.. Nature.

[49] Tarun AS, Peng X, Dumpit RF, Ogata Y, Silva-Rivera H (2008). A combined transcriptome and proteome survey of malaria parasite liver stages.. Proc Natl Acad Sci U S A.

[50] Young JA, Fivelman QL, Blair PL, de la Vega P, Le Roch KG (2005). The Plasmodium falciparum sexual development transcriptome: a microarray analysis using ontology-based pattern identification.. Mol Biochem Parasitol.

[51] Bozdech Z, Zhu J, Joachimiak MP, Cohen FE, Pulliam B (2003). Expression profiling of the schizont and trophozoite stages of Plasmodium falciparum with a long-oligonucleotide microarray.. Genome Biol.

[52] Bohlander SK, Espinosa R, Le Beau MM, Rowley JD, Diaz MO (1992). A method for the rapid sequence-independent amplification of microdissected chromosomal material.. Genomics.

