# Supplementary figures and images for: Histone Deacetylases Play a Major Role in the Transcriptional Regulation of the Plasmodium falciparum Life Cycle
Source: PLoS Pathog. 2010 Jan 22;6(1):e1000737. doi: 10.1371/journal.ppat.1000737 (PMC2809759; doi:10.1371/journal.ppat.1000737)

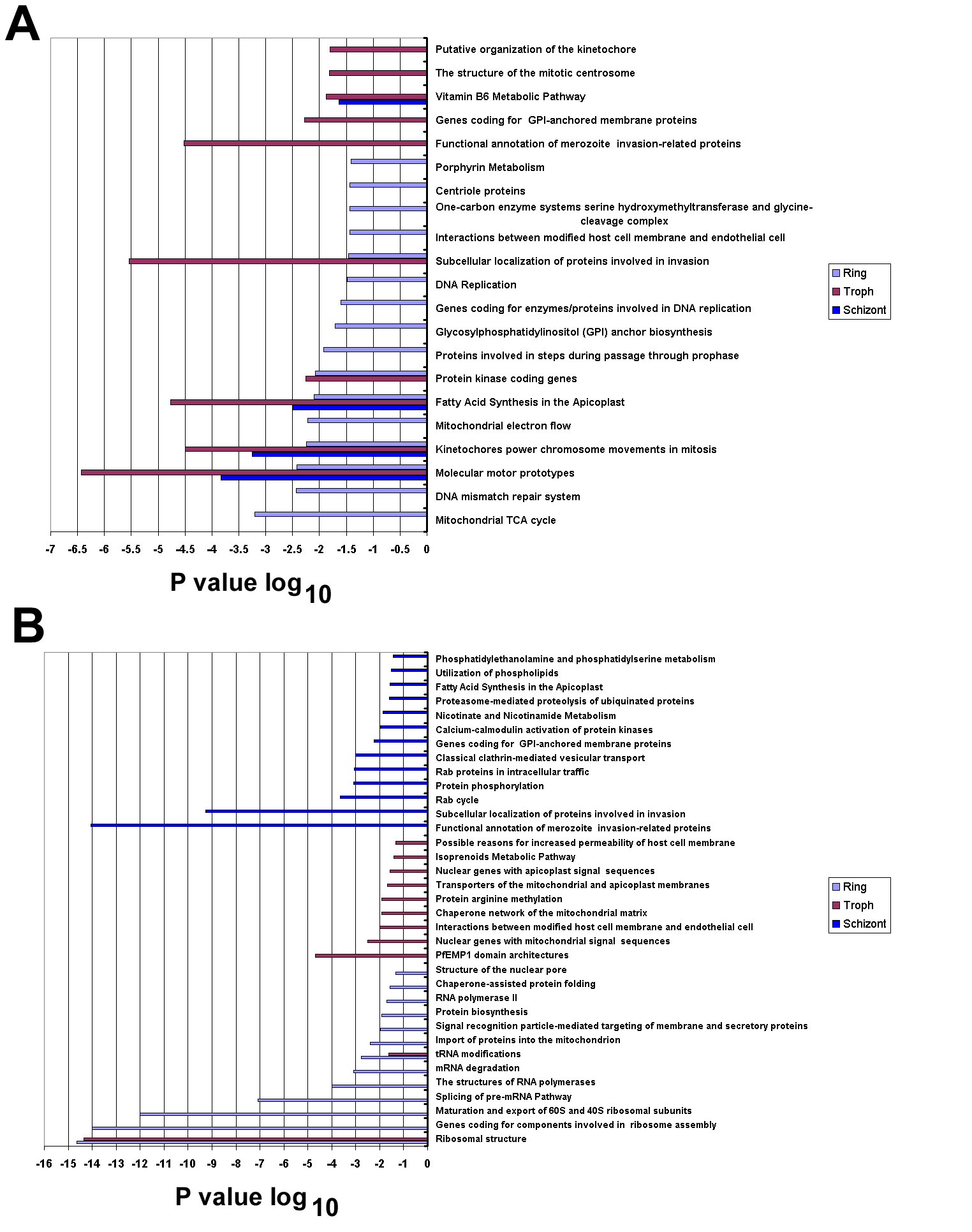

Supplement: Figure S1 — Functional classes affected by apicidin treatment. Functional groups significantly associated (P value <0.05) with induced (A) and repressed (B) expression (>2-fold) by apicidin in all three stages of the P. falciparum IDC are shown. The functional annotations based on Malaria Parasite Metabolic Pathways (MPMP) are used. The P values are shown as logarithm values with base 10. (0.39 MB TIF) [file ppat.1000737.s002.tif]

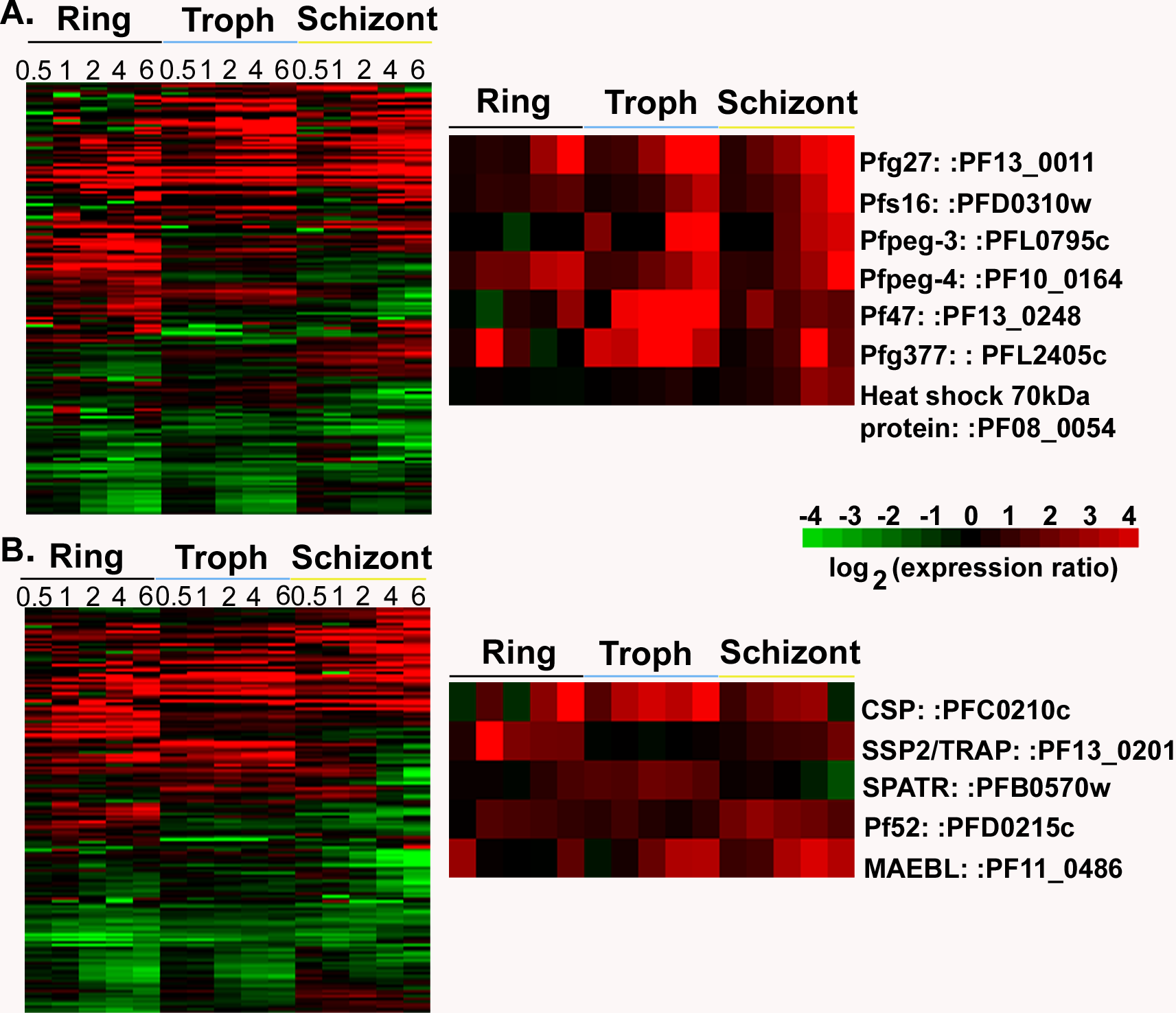

Supplement: Figure S2 — Apicidin treatment alters expression of gametocyte (A) and sporozoite (B) genes in asexual blood stages. Highly synchronized P. falciparum cells: rings (6–14 hpi), trophozoites (20–28 hpi) and schizonts (34–42 hpi) were treated with 70nM apicidin. RNA samples were collected at 0.5, 1, 2, 4 and 6 hours post treatment. cDNA, synthesized from the RNA samples, was labeled with Cy5 and hybridized against the Cy3 labeled 3D7 reference pool. The data include mRNA abundance ratios between each time point sample and the 3D7 reference pool. The data was filtered as described in material and methods. The first 500 genes showing the highest mRNA abundance in the publicly available P. falciparum gametocyte and sporozoite gene expression datasets [2] were analyzed within our apicidin perturbation dataset. Genes with a 2 or greater fold difference in expression, in at least one time point, in apicidin treated rings, trophozoites or schizonts were shown. (0.62 MB TIF) [file ppat.1000737.s003.tif]
